# Switching patients with inflammatory arthritis from Etanercept (Enbrel^®^) to the biosimilar drug, SB4 (Benepali^®^): A single-centre retrospective observational study in the UK and a review of the literature

**DOI:** 10.31138/mjr.30.1.69

**Published:** 2019-05-31

**Authors:** Anastasia-Vasiliki Madenidou, Andrew Jeffries, Sneha Varughese, Stephen Jones, Hanadi Sari-Kouzel, Helen Veevers, Chandini Rao

**Affiliations:** 1 Rheumatology Department, Blackpool Teaching Hospitals NHS Foundation Trust, Blackpool, United Kingdom,; 2 Centre for Rheumatology, University College London, London, United Kingdom

**Keywords:** arthritis, biologics, biosimilars, etanercept, SB4

## Abstract

**Objective/Aim:**

SB4 (Benepali^®^), the Etanercept biosimilar, is licenced in the UK for the same indications as the reference product, Enbrel^®^. In 2016, the Rheumatology Department at Blackpool Teaching Hospitals switched the Etanercept patients, who gave consent, to SB4. A proportion of these patients switched back to Etanercept and therefore we aimed to investigate the reasons of SB4 withdrawal and compare our results with the current evidence.

**Methods:**

We included all the patients switched to SB4 until April 2018, identified from the departmental biologics database. We also searched the published and grey literature through November 2018 for similar articles.

**Results:**

72 Etanercept patients switched to SB4, of which 19 (26.4%) switched back to Etanercept within 6 months on the biosimilar product. All the 19 patients remained on Etanercept until the time of data analysis. The main reason of withdrawal was loss of effect (LOE, 58%). In RA, the duration on Etanercept was associated with SB4 withdrawal (OR 1.43 [95% CI 1.02, 2.00]) and LOE was reflected in the DAS- 28, PGS and CRP increase and in the number of tender joints (all p <0.05). We found ten observational studies reporting 3184 patients, who switched from Etanercept to SB4 and 432 of them (14%) stopped SB4.

**Conclusion:**

The majority (73.6%) stayed on SB4, which is consistent with the current evidence. Taking also into consideration the results of the other studies, it is unclear if this withdrawal is a true failure on SB4, nocebo effect or spontaneous disease flare.

## INTRODUCTION

Etanercept (Enbrel^®^) is a tumour necrosis factor (TNF) antagonist used in the treatment of several inflammatory diseases, such as rheumatoid, juvenile idiopathic and psoriatic arthritis, ankylosing spondylitis and non-radio-graphic axial spondyloarthropathy. In February 2016, a biosimilar formulation of etanercept, SB4 (Benepali^®^) was launched in the United Kingdom (UK).^1^

To gain approval in the European Union (EU), biosimilar medicines must demonstrate that they are as safe and as effective as the originator, and have the same quality characteristics. A comprehensive study demonstrated that SB4 is highly similar to the reference product (Enbrel^®^) in terms of structure, physicochemical characteristics, and biological activity.^2^ A phase III, randomized, double-blind study comparing SB4 with Enbrel^®^ in patients with moderate to severe rheumatoid arthritis despite methotrexate therapy concluded that the efficacy was comparable between the two groups with a similar safety profile.^3^

Based on these data, the Agency’s Committee for Medicinal Products for Human Use (CHMP) decided the approval of SB4 to all indications for which the reference product, Etanercept is approved and therefore the EMA (European Medicines Agency) recommended its authorization in the European Union.^4^ On 1^st^ April 2016, a new CMU (Commercial Medicines Unit) contract started in the UK with price for SB4 10% below that of the reference product.^1^

On this background, in 2016 the Rheumatology Department at Blackpool Teaching Hospitals contacted all patients receiving (Enbrel^®^) by letter to inform them of the possibility of switching to SB4. A series of patient information meetings were held, informing patients on Enbrel^®^ about the cost benefits of switching to SB4. Patients were also advised they could switch back to Enbrel^®^, if they experienced any adverse effects or loss of effect (LOE). Patients who could not attend the group information meetings were counselled about the switch during their next routine clinical appointment. Only patients who gave verbal consent were switched.

Although the aforementioned studies’ results ensure that switching patients from Enbrel^®^ to SB4 has significant cost benefits without any efficacy or safety issues, we realized in clinical practice that a proportion of our patients switched back to the reference product (Enbrel^®^). Therefore, we decided to look through the clinical letters of our patients and investigate which were the reasons of SB4 withdrawal, if there are any baseline associated factors, if the LOE is reflected in the objective disease activity measures and compare our results with the current evidence.

## MATERIALS AND METHODS

We report our observational study in accordance to the Strengthening the Reporting of Observational Studies in Epidemiology (STROBE) statement.^5^

We included all the rheumatology patients switching from Enbrel^®^ to SB4 until April 2018. The patients were identified from the departmental biologics database. Data were collected from the patients’ records (clinical letters, case notes and electronic record).

Switchers are the patients that changed from Enbrel^®^ to SB4. Non-switchers are the patients that maintained on Enbrel^®^ and did not change to SB4. Back-switchers are the switchers, who stopped SB4 and restarted Enbrel^®^ during the follow-up period. Retention rate is the proportion of patients who stayed on the same medication and on the other hand, withdrawal rate is the proportion of patients that stopped their medication during the follow-up period.

All data were analysed descriptively. Baseline characteristics associated with SB4 withdrawal were explored by multivariate logistic regression analysis stratified by diagnosis (rheumatoid arthritis [RA], axial spondyloarthropathy [SpA], psoriatic arthritis [PsA]). The characteristics included age, gender, duration of disease, co-treatment with DMARDs, concomitant Methotrexate, number of biological DMARDs (bDMARDs) before Enbrel^®^, duration on Enbrel^®^, number of swollen and tender joints, baseline ESR and CRP; only for RA: seropositivity for RF and/or anti-CCP, DAS-28 before any bDMARD, baseline DAS-28 and Patient Global Score (PGS); only for SpA: HLA- B27 status, Bath Ankylosing Spondylitis Disease Activity Index (BASDAI) before any bDMARD, baseline BASDAI and pain Visual Analogue Score (VAS) and only for PsA: Baseline Patient (PtGA) and Physician Global Assessment (PGA).

Wilcoxon signed-rank test was performed to compare the various expressions of disease activity (DAS- 28 and PGS for RA, BASDAI and pain VAS for SpA, PtGA and PGA for PsA, ESR, CRP, swollen and tender joints) before switching to SB4 and before SB4 withdrawal in patients with loss of effect.

For the literature review, we searched MEDLINE, Em-base and the Cochrane Central Register of Controlled Trials without any language limitations from inception to November 2018. We also searched the abstracts of BSR (British Society for Rheumatology), EULAR (European League Against Rheumatism) and ACR/ARHP (American College of Rheumatology/Association of Rheumatology Health Professionals) meetings from 2016 to 2018, since the SB4 was given a marketing authorization in 2016. The search was based on the following keywords: Be-nepali^®^, SB4, Etanercept, Enbrel^®^ and arthritis. Studies reporting on patients switching from Enbrel^®^ to SB4 were selected for inclusion.

## RESULTS

72 out of 104 (69.2%) patients on Enbrel^®^ switched to SB4 at Blackpool Teaching Hospitals NHS Foundation Trust. The study duration was 19 months (interquartile range [IQR] 17- 20). Baseline characteristics of switchers are presented in *Table 1*.

**Table 1:** Baseline characteristics of patients switching from Etanercept (Enbrel^®^) to SB4 (Benepali^®^) at Blackpool Teaching Hospitals NHS Foundation Trust

	**Rheumatoid arthritis**	**Axial Spondyloarthropathy**	**Psoriatic arthritis**
Number, n	36	23	13
Female, n (%)	27 (75%)	7 (30%)	6 (46%)
Age, years	62 (56–77)	56 (46–66)	54 (51.5–67.5)
Duration on Etanercept before switching to SB4, years	3.8 (2.3–7.5)	5.2 (3.1–7.5)	2.8 (2.3–6.6)
Duration on Etanercept of patients in clinical remission before switching to SB4, years	5.4 (4.3–7.1)	5.7 (2.9–7.2)	3.3 (2.5–6.4)
Duration of disease before switching to SB4, years	15 (8.2–21)	29 (17.3–38)	17 (12.5–26.5)
Co- treatment with DMARDs, n (%)	32 (89%)	2 (8,7%)	9 (69.2%)
Concominant Methotrexate, n (%)	26 (72%)	1 (4,3%)	6 (46.2%)
On other bDMARDs before Etanercept, n (%)	8 (22.2%)	4 (17.4%)	2 (15.4%)
DAS- 28 before any bDMARD	6.18 (5.72–6.71)	NA	NA
DAS- 28 before switching to SB4	2.87 (1.89–3.73)	NA	NA
BASDAI before any bDMARD	NA	7.01 (6.25–8.68)	NA
BASDAI before switching to SB4	NA	2.95 (1.94–4.85)	NA
Number of tender joints, n	1.00 (0.00–4.00)	0.00 (0.00–0.00)	1.00 (0.00–2.50)
Number of swollen joints, n	1.00 (0.00–2.00)	0.00 (0.00–0.00)	1.00 (0.00–2.00)
ESR before switching to SB4, mm/hr	12.50 (6.00–19.75)	6.00 (2.00–9.00)	7.00 (5.00–26.50)
CRP before switching to SB4, mg/dl	2.00 (1.00–5.00)	4.00 (1.00–7.90)	2.00 (1.00–9.50)

Numbers are medians (interquartile ranges). bDMARDs= biological DMARDs, NA= Not Applicable

19 (26.4%) SB4 patients switched back to Enbrel^®^ within 6 months (IQR 3.5–10) on the biosimilar product. 12 out of 19 patients had rheumatoid arthritis, 5 axial SpA and 2 PsA. The reasons of withdrawal were LOE (58%), adverse events (32%), infection (5%) and difficulty using the pen device (5%). The reported adverse events were headache, dyspnoea, weight gain, hair loss, rash and fatigue. In RA, the duration on Enbrel^®^ was associated with SB4 withdrawal (OR 1.43 [95% CI 1.02, 2.00]) and no statistically significant factors were found in SpA and PsA.

For RA, LOE is reflected in the DAS- 28 increase (2.99), PGS increase (40 mm), increase in tender joints (3.5) and CRP increase (2 mg/dl) (all p <0.05), but not in the number of swollen joints and ESR. In SpA and PsA, there were no statistically significant changes in the disease activity measures.

All the 19 patients remained on Enbrel^®^ until the time of data analysis (follow- up period: 12 months [IQR 7.5–15.5]). Ten observational studies (3184 patients) reporting results about switching from Enbrel^®^ to SB4 in arthritis were identified with our search strategy, 2 published articles^6,7^ and 8 conference abstracts.^8–15^ All of them were conducted in Europe and the majority of them (70%) in the UK. The duration of studies ranged from 3 to 12 months. The Danish study was the largest one with 1621 patients.^6^ The baseline characteristics of the included studies are presented in *Table 2*.

**Table 2:** Baseline characteristics and results of the studies included in our review

**Author, Year**	**Study duration, months**	**Disease arms**	**Patients switched to SB4, n (%)**	**Female, n (%)**	**Age, years**	**Etanercept duration before switching to SB4, years**	**Concomitant MTX, n (%)**	**On other bDMARDs before Etanercept, n (%)**	**DAS- 28 before switching to SB4**	**BASDAI before switching to SB4**	**Patients failed on SB4, n (%)**	**Reasons of failure on SB4**	**Patients switched to another bDMARD**	**Patients switched back to Etanercept**	**Patients stayed on Etanercept after switching back**
Glintborg 2018 (6)	12	RA	933 (58%)	689 (74%)	61 (49 to 70)	NR	556 (60%)	442 (47%)	1.9 (1.3 to 2.8)	NA	194 (21%)	LOE (46%), AEs (26%), other reasons (18%)	104/299 (35%)	120/299 (40%)	104/120 (87%)
		AxSpA	337 (20,8%)	115 (34%)	48 (39 to 57)	NR	51 (15%)	214 (64%)	NA	3.3 (1.5 to 5.2)	52 (15%)				
		PsA	351 (22%)	160 (46%)	52 (43 to 61)	NR	168 (48%)	170 (48%)	NA	1.8 (1.1 to 2.4)	53 (15%)				
Tweehuysen 2018 (7)	6	RA	433 (69%)	341 (55%)	57	3 (2 to 6)	NR	NR	1.9 (1.5 to 2.6)^*^	NA	60 (10%)	LOE (43%), AEs (47%), other reasons (10%)	32/60 (53%)	17/60 (28%)	NR
		AxSpA	64 (10%)							3.1 (1.8 to 5.4)					
		PsA	128 (21%)							NA					
Alkoly 2018 (14)	6	RA	87 (55%)	70 (80.5%)	62	1.3 (0.8 to 2.2)	NR	6 (6.9%)	2.82	NA	14 (8.9%)	LOE (50%), AEs (50%)	0	14/14 (100%)	14/14 (100%)
		AxSpA	41 (26%)	7 (17%)	42	1 (0.4 to 1.6)		3 (7.3%)	NA	3.27					
		PsA	30 (19%)	12 (40%)	55	2.1 (1.6 to 2.1)		1 (3.3%)	NA	NA					
Dybal 2017 (15)	NR	RA	38 (100%)	NR	NR	NR	NR	NR	3.08	NA	6 (16%)	LOE (67%), AEs (33%)	0	5/6 (83%)	NR
		AxSpA	0						NR						
		PsA	0						NR						
Krueger 2018 (8)	3	RA	163 (64%)	112 (69%)	60.8 (54 to 69)	NR	NR	NR	2.0	NA	NR	NR	NR	NR	NR
		AxSpA	92 (36%)	28 (30.4%)	50.7 (40 to 61)				NA	3.0					
		PsA	0	NA	NA				NA	NA					
Lee 2018 (9)	8	RA	56 (100%)	NR	NR	NR	NR	NR	NR	NR	9 (16%)	LOE (89%), AEs (11%)	7/9 (78%)	2/9 (22%)	NR
		AxSpA													
		PsA													
Ma 2018 (10)	6	RA	32 (64%)	23 (72%)	60	6	NR	NR	NR	NR	8 (16%)	LOE (40%), AEs (50%), other reasons (10%)	NR	NR	NR
		AxSpA	15 (30%)	1 (7%)											
		PsA	3 (6%)	NR											
Rabbits 2017 (11)	NR	RA	44 (100%)	NR	NR	NR	NR	NR	NR	NR	8 (18%)	LOE (63%), infection or planned surgery (37%)	NR	NR	NR
		AxSpA													
		PsA													
Rajamani 2018 (12)	NR	RA	120 (100%)	NR	NR	9.2	96 (80%)	15 (13%)	NR	NR	18 (15%)	LOE (60%), AEs (40%)	NR	9/18 (50%)	NR
		AxSpA													
		PsA													
Smith 2018 (13)	NR	RA	217 (100%)	NR	NR	NR	NR	NR	NR	NR	10 (4.6%)	LOE (40%), AEs (50%), missing data (10%)	0	10/10 (100%)	10/10 (100%)
		AxSpA													
		PsA													

Numbers are medians (interquartile ranges).

* for both RA and PsA

MTX= Methotrexate, bDMARDs= biological DMARDs, RA= rheumatoid arthritis, PsA= Psoriatic arthritis, AxSpA= axial Spondyloarthropathy, LOE= Loss of effect, AEs= adverse events, NR= Non Reported, NA= Non Applicable,

Overall, 3184 patients changed from Enbrel^®^ to SB4 and 432 (14%) failed on SB4 with the percentage in each study ranging from 4.6% to 18%. Loss of effect and adverse events were the most common reasons for withdrawal. The patients failing on SB4 switched back to Enbrel^®6,9,12–15^or changed to another bDMARD.^6,7,9^ The results of each study are presented in *Table 2*.

We present in *Table 3* the adverse events for withdrawal in SB4 in six out of ten studies.^6,7,12–15^ Four studies did not report the range of adverse events.^8–11^ The most common adverse events were rash/itching (15.6%), infections (8.5%), headache/migraine (8.5%) and local injection problems (7.8%). One additional adverse event (weight gain) is reported in our study, but not in the literature.

**Table 3:** Adverse events for withdrawal in SB4 (Benepali^®^) switchers reported in the studies included in our review

**Adverse events**	**Number of events(n= 141)**
Anxiety	1 (0.7%)
Arthralgia	10 (7%)
Bladder dysfunction	1 (0.7%)
Bruising	2 (1.4%)
Chest pain	4 (2.8%)
Coughing	2 (1.4%)
Diarrhea	4 (2.8%)
Dizziness	5 (3.5%)
Dizziness, nausea, headache, loss of appetite	4 (2.8%)
Dyspnea	3 (2.1%)
Erectile dysfunction	1 (0.7%)
Fatigue	8 (5.7%)
Fever	2 (1.4%)
Hair loss	2 (1.4%)
Headache/migraine	12 (8.5%)
Hyperhidrosis	2 (1.4%)
Hypertension	1 (0.7%)
Hypotension	1 (0.7%)
Increased ALT level	1 (0.7%)
Infections	12 (8.5%)
Leg cramps	2 (1.4%)
Leucopenia or neutropenia	4 (2.8%)
Local injection problems	11 (7.8%)
Mood disturbances	1 (0.7%)
Mouth or/and skin ulceration	2 (1.4%)
Myalgia	3 (2.1%)
Nausea	7 (5%)
Neuropathies	1(0.7%)
Palpitations	2 (1.4%)
Paresthesia	2 (1.4%)
Psoriasis worsening or pustulosis	2 (1.4%)
Rash/itching	22 (15.6%)
Uveitis	1 (0.7%)
Visual disturbance	3 (2.1%)

In the Danish study,^6^ multiple Cox proportional hazards regression analyses stratified by diagnosis (RA or PsA or axial SpA) were conducted to estimate withdrawal rates adjusted for clinically relevant variables. They found that the RA patients who stopped SB4, did not take Methotrexate and had higher PGS. In PsA, associated factors were female gender, higher PtGA and lower SB4 doses. No significant factors were found in axial spondyloarthropathy. They also compared their results with the patients that did not switch from Enbrel^®^ to SB4 (non-switchers) and with a historic cohort of patients on Enbrel^®^ with start date 1^st^ January 2015. One-year adjusted retention rates were: non-switchers: 77%/switchers: 83%/historic cohort: 90%.

In the Dutch study, they also compared the switchers to a historical cohort with patients on Enbrel^®^ from 2014. They found out that the crude treatment persistence rate for biosimilar SB4 over 6 months was 90%, compared to a 6-month treatment persistence rate of 92% for originator Enbrel^®^.

## DISCUSSION

In our observational study, the majority of switchers (73.6%) stayed on the biosimilar product. The main reasons of withdrawal were LOE (58%) and adverse events (32%). In RA, LOE is reflected mainly in the subjective disease activity measures, except for CRP. Interestingly, all the patients switching back to the originator stayed on this treatment during the follow-up period.

19 (26.4%) SB4 patients switched back to Enbrel^®^, 12 with RA, 5 with axial SpA and 2 with PsA. Comparing the back- switchers with the initial number of patients in each disease group, 33% of the RA patients, 22% with SpA and 15% with PsA switched back to Enbrel^®^. The discrepancy in retention rates among the disease groups is also reported in the Danish study with the lowest retention rate in patients with RA.^6^ The overall retention rate is only reported in the other articles.

Nocebo effect is defined as the incitement or the worsening of symptoms induced by any negative attitude from non-pharmacological therapeutic intervention, sham, or active therapies. The nocebo effect in switching from an original to a biosimilar has been recently described in the literature.^16^ We tried to minimize the impact of nocebo effect in our patients by only switching patients from Enbrel^®^ to SB4, if they had given consent. However, in other hospitals they changed all the patients to the biosimilar product.^9,11^ Two of our findings may suggest that SB4 withdrawal is not a real failure, but nocebo effect. We found that in RA, the duration on Enbrel^®^ was associated with SB4 withdrawal. In other words, patients that had been longer on the originator may be more anxious about a change in their treatment. Additionally, the patients with SB4 withdrawal had statistically significant increase mainly in the subjective measures (DAS- 28, PGS, tender joints) and only in one objective measure, the CRP.

Our SB4 withdrawal percentage (24%) was higher than the reported in the literature (ranging from 4.6% to 18%),^6–15^ but our study had the longest follow-up period, 19 months (IQR 17–20). In accordance with this, our withdrawal percentage (24%) was close to the study with the second longest follow-up (1 year) at 18%.^6^ The above results may suggest that if a patient is longer on SB4, they are more likely to fail on SB4. The question is if this a true failure on SB4 or a spontaneous disease flare after a long duration on the same treatment. In favour of the second hypothesis, are the similar retention rates on SB4 and on Enbrel^®^ in the Danish and Dutch study.^6,7^ This may mean that patients who failed on SB4 would also have failed on Enbrel^®^, if they had stayed on it. However, SB4 withdrawal rate is scrutinized under the light of the switching process. It would be interesting to compare the withdrawal rates of switchers on SB4 and patients starting on SB4 de novo, and this is the aim of our upcoming study.

There is not a uniform strategy for the patients failing on SB4. We decided to switch these patients back to Enbrel^®^. Three studies do not present any data about patients experiencing side effects or joint flare on SB4.^8,10,11^ The rheumatology department of Kings College Hospital NHS Foundation Trust changed the majority to a third biologic and the minority back to Enbrel^®^ without reporting the retention rate.^9^ The Danish study reported that approximately half the patients switched to another biologic and the rest back to Enbrel^®^.^6^ The Dutch study changed 53% of the patients to another bDMARD, 28% to Enbrel^®^ and 18% continued without a bDMARD.^7^ Four studies reported switching all the patients back to Enbrel^®^.^12–15^

The Danish study reported that 87% of the back- switchers were still on Enbrel^®^ with median treatment duration of 236 (155 to 302) days, which is consistent with our 100% retention rate. Rotherham hospital’s experience is similar to ours with all adverse events resolving and joint flares settled or settling after switching back to Enbrel^®^^13^ and all the patients from Barts also achieved improvement after switching back to Enbrel^®^.^14^ Based on these data, we suggest switching back to the originator, rather than moving to another biologic, when SB4 fails.

According to the Danish study, the patients not in remission before starting on SB4, had lower retention rates than the patients in remission.^6^ This is not confirmed in our study with 37.5% of the patients that stayed on SB4 being in baseline remission, in contrast with 77.8% of the patients that failed on SB4.

The Danish study reports that according to cox regression analysis, the RA patients, who stopped SB4, did not take Methotrexate. The phase III study that contributed to the approval of SB4 for the same indications as the originator, randomized patients with moderate to severe RA despite methotrexate treatment to SB4 and Enbrel^®^ and concluded that the efficacy and safety of SB4 was comparable between the two medications. However, this means that SB4 and Enbrel^®^ were compared on the background of Methotrexate and the aforementioned association of the Danish study suggests that SB4 may not be comparable to Enbrel^®^ under different circumstances. Strengths and limitations should be acknowledged, when interpreting our results. The study reports real world data for patients treated in routine care and patients act as their own controls in the evaluation of disease activity. We studied all the patients that changed to SB4 in order to minimize the selection bias and to provide information through the whole period of switching our patients to SB4. On the other hand, the observational study design suggests associations rather than causal relationships. Another limitation is our small sample size.

In conclusion, we found that a single-centre switch from Enbrel^®^ to biosimilars in 72 patients with inflammatory arthritis had a 73.6% retention rate, suggesting that SB4 is effective and safe in the clinical practice. However, it is not clear if the LOE is true failure on SB4, spontaneous disease flare or nocebo effect, despite the efforts of minimizing the latter. Interestingly, all the patients switching back to Enbrel^®^ maintained the originator, which is also supported by other studies. On the background of the emerging biosimilars, we suggest that future studies, ideally with large sample size, should focus on the patients failing on biosimilars and investigate which patients are likely to fail on them. We acknowledge the cost benefits of switching patients from originators to biosimilars, but we should also pay attention to the costs of an unsuccessful switching process (sick leave due to LOE or adverse events, additional rheumatology reviews and cost of unused biosimilars).
